# Combining Climatic Projections and Dispersal Ability: A Method for Estimating the Responses of Sandfly Vector Species to Climate Change

**DOI:** 10.1371/journal.pntd.0001407

**Published:** 2011-11-29

**Authors:** Dominik Fischer, Philipp Moeller, Stephanie M. Thomas, Torsten J. Naucke, Carl Beierkuhnlein

**Affiliations:** 1 Department of Biogeography, University of Bayreuth, Bayreuth, Germany; 2 Division of Parasitology, Department of Zoology, University of Hohenheim, Stuttgart, Germany; 3 Parasitus Ex e.V., Niederkassel, Germany; 4 Laboklin GmbH & Co.KG, Bad Kissingen, Germany; Universidad de Buenos Aires, Argentina

## Abstract

**Background:**

In the Old World, sandfly species of the genus *Phlebotomus* are known vectors of *Leishmania*, *Bartonella* and several viruses. Recent sandfly catches and autochthonous cases of leishmaniasis hint on spreading tendencies of the vectors towards Central Europe. However, studies addressing potential future distribution of sandflies in the light of a changing European climate are missing.

**Methodology:**

Here, we modelled bioclimatic envelopes using MaxEnt for five species with proven or assumed vector competence for *Leishmania infantum*, which are either predominantly located in (south-) western (*Phlebotomus ariasi*, *P. mascittii* and *P. perniciosus*) or south-eastern Europe (*P. neglectus* and *P. perfiliewi*). The determined bioclimatic envelopes were transferred to two climate change scenarios (A1B and B1) for Central Europe (Austria, Germany and Switzerland) using data of the regional climate model COSMO-CLM. We detected the most likely way of natural dispersal (“least-cost path”) for each species and hence determined the accessibility of potential future climatically suitable habitats by integrating landscape features, projected changes in climatic suitability and wind speed.

**Results and Relevance:**

Results indicate that the Central European climate will become increasingly suitable especially for those vector species with a current south-western focus of distribution. In general, the highest suitability of Central Europe is projected for all species in the second half of the 21st century, except for *P. perfiliewi*. Nevertheless, we show that sandflies will hardly be able to occupy their climatically suitable habitats entirely, due to their limited natural dispersal ability. A northward spread of species with south-eastern focus of distribution may be constrained but not completely avoided by the Alps. Our results can be used to install specific monitoring systems to the projected risk zones of potential sandfly establishment. This is urgently needed for adaptation and coping strategies against the emerging spread of sandfly-borne diseases.

## Author Summary

Growing evidence exists on the emergence of sandfly-borne diseases in the light of climate change. Determining the principle responses of phlebotomine sandflies to climatic changes supports our understanding of future regions that will be threatened by new-establishments of this important group of disease vectors. The aim of this paper is to combine projected climatic suitability for five *Phlebotomus* species in Central Europe (Austria, Germany and Switzerland) for different time-periods during the 21st century with their potential spreading capacity to disperse to climatically suitable areas. We indicate that the Central European climate will develop toward the preferred bioclimatic niche of the species, especially from mid-century onwards. Nevertheless, we also elucidate within this study that sandflies will hardly be able to occupy the whole areas that will provide suitable climatic conditions due to their limited natural dispersal ability. Our approach provides a framework to combine statistical modelling techniques with expert knowledge on species ecology. Indications of future occurrences of disease vectors may help to initiate surveillance systems in specific regions at an early stage of risk exposure. Hence, the threat of the climate-driven spatial extension of disease vectors and consequently of potentially emerging vector-borne diseases can be counteracted.

## Introduction

Globally, the number of vector-borne infections in humans and animals increases rapidly, meanwhile causing almost one third of all cases of emerging infectious diseases [1]. In the Old World, sandfly species of the genus *Phlebotomus* serve as vectors for sandfly-borne pathogens such as *Leishmania*, *Bartonella* and several viruses (e.g. *Phlebovirus*, *Vesiculovirus* and *Orbivirus*) [2]–[4]. Sandfly-borne diseases and in particular visceral leishmaniasis are a main public health concern [5], which demands more attention in science and policy [6]. While the spatial distribution of leishmaniasis seems to expand in southern parts of Europe [7], [8], first cases of autochthonous origin are recently reported from Central Europe [9]–[11], where this disease was not endemic in the past.

The presence of sandflies as vectors is mainly regulated by the species' climatic requirements on temperature and humidity or soil moisture, respectively [3], [12]–[15]. Temperature and humidity are also the main factors impacting the altitudinal structure of sandfly occurrences [16]. It is known that sandflies react very sensitive to wind speed and prefer breeding sites sheltered from wind [17]–[20]. Beyond that, high wind speed decreases or even excludes flight activity [17], [21]. For the purpose of inferring geographic distribution for sandflies, the advantages of ecological niche models have been demonstrated on the example of *Lutzomyia* species (*Lutzomyia spp.*) in the New World [22]. For the first time, Peterson and Shaw [23] integrated climate change scenarios in order to project future distribution of *Lutzomyia spp.* in Brazil. Recently, range expansions for sandflies of the genus *Lutzomyia* have also been projected for North America in the face of climate change [24].

For Europe, surprisingly, only few studies estimated the risk of potential range expansions of sandflies in the face of climate change (e.g. [25], [26]). The need for such studies is supported by the first sandflies catches in Central Europe. *P. mascittii* has been caught in Austria on the frontier to Slovenia [27]. Furthermore, *P. mascittii* is reported from the “Upper Rhine Valley” in the outermost southwest of Germany near the French border [28]. *P. perniciosus* seems to be established in the German state of “Rhineland-Palatinate” [29]. These findings may either indicate spreading tendencies from Mediterranean regions or range expansion from small Central European refugial areas, which may have already been occupied by the species during the Holocene climate optimum about 6,500 years ago [30]. Possibly, sandflies has occupied more areas in the past than it was noticed. For Austria, establishment of sandflies in formerly non-endemic areas can be expected already by moderately increasing temperatures in the 21^st^ century [25]. Recently, Fischer et al. [26] estimated potential temperature-derived establishment of sandflies in Germany by transferring the required temperature during their activity phase and annual mean temperature for persistence to the expected future climate conditions in Germany using data of a spatio-temporal highly resolved regional climate model. But up to now, projections of the current and climate-driven potential future distribution of *Phlebotomus spp.* which additionally consider species-specific dispersal ability are missing. As climate is expected to change rapidly in the 21^st^ century, sandflies are forced to react promptly. Here, we close this gap of knowledge and hypothesise:

1. Climatic suitability for *Phlebotomus spp.* in Central Europe will generally increase within the 21^st^ century. Expectedly, the climatic requirements for sandflies with current (south-) western European regions of distribution are supposed to be fulfilled in the south-westernmost parts of Central Europe in the 21^st^ century. Instead, species with a south-eastern focus of distribution are thought to find favourable conditions in the south-easternmost Central European regions.
2. Species with current (south-) western focus of distribution will spread north-eastwards as they are not hampered by natural dispersal barriers. Instead, the Alps will restrict a direct range expansion for species that are currently distributed in the (south-) east of Europe.

## Materials and Methods

### Bioclimatic envelope modelling for sandfly species

#### Species presence records and climatic variables

Documented presence records of *Phlebotomus spp.* were taken from literature. Most of the occurrence data were provided by Artemiev and Neronov [31]. This was done by digitising their analogue maps of presence records. Additional presence records were taken from peer-reviewed articles by searching within the literature databases ISI Web of Science, MEDLINE and BIOSIS Previews from 1984 onwards. The number of presence records is listed in Table 1.

**Table 1:** Number of species presence records, AUC-values, and training gain for the selected bioclimatic variables.

	*P. ariasi*	*P. mascittii*	*P. perniciosus*	*P. neglectus*	*P. perfiliewi*
Presence records	79	66	273	90	124
AUC (Training data)	0.94	0.97	0.92	0.92	0.96
AUC (Test data)	0.93	0.93	0.90	0.89	0.95

Model training gain for variables used in isolation (upper value), and for the remaining data set if the specific variables is dropped from the set (lower value)						
BIO 10	Mean temperature of the warmest quarter	1.50	1.91	1.18	0.98	0.81
		0.51	0.57	0.65	0.16	0.10
BIO 11	Mean temperature of the coldest quarter	1.35	1.74	1.30	0.70	0.87
		1.15	1.24	0.67	0.67	0.49
BIO 12	Annual precipitation	-	1.94	-	-	0.88
		-	0.95	-	-	0.24
BIO 13	Precipitation of the wettest month	-	-	1.35	-	-
		-	-	0.73	-	-
BIO 16	Precipitation of the wettest quarter	-	-	1.36	-	-
		-	-	0.70	-	-
BIO 17	Precipitation of the driest quarter	1.43	1.88	-	0.97	-
		0.61	0.78	-	0.29	-
BIO 18	Precipitation of the warmest quarter	1.45	-	-	0.98	0.86
		0.73	-	-	0.32	0.32
BIO 19	Precipitation of the coldest quarter	1.38	1.85	1.29	0.94	0.93
		0.73	0.78	1.00	0.43	0.33

AUC-values are a threshold-independent model quality criterion and range from 0 to 1 (perfect fit). Useful models yielded in AUC-values above 0.7, where excellent models achieve at least AUC-scores above 0.9. Training gains are determined by Jackknife test for the selected variables by using only this variable for the model (upper value) and if the specific variable is removed for the rest of the variable set (lower value). For the species with current (south-) western focus of distribution (*P. ariasi*, *P. mascittii* and *P. perniciosus*) BIO 11 (Mean temperature of the coldest quarter) represents the most important variable. This is indicated by the highest training gain of the model by using only this variable and the lowest training when this variable is removed from the set of variables. The drop of BIO 10 (Mean temperature of the warmest quarter) from the set of variables instead seems to lower training gain most for the species with (south-) eastern focus of distribution. BIO 13 (Precipitation of the wettest month) is identified as most important variable when used in isolation for *P. neglectus*, while BIO 11 seems to be most influencing factor in isolation regarding the occurrences of *P. perfiliewi*.

Current bioclimatic data were taken from http://www.worldclim.org [32] in 5 Arcmin resolution (approximately 10 km grid size for Central Europe). Higher spatial resolution would not correspond to the spatial accuracy of occurrence data for sandfly species. We defined all regions that contain our presence records (Europe, northern Africa and the Middle East) as climatic background, where our models were trained. Selection of the most important bioclimatic variables was done via Jackknife test. We considered results of the Jackknife tests for the model training gain for all variables in isolation and for the remaining set of variables when the isolated variable is dropped from the set [33]. To reduce collinearity in the data set [34] those variables that had a Pearson correlation coefficient r>0.7 with any other higher-ranking variable in the results of the Jackknife test variables were removed. We applied the variable selection procedure separately for each species.

The high-resolution regional climate model COSMO-CLM (CCLM) was applied for future projections in Europe. This dynamically downscaled model is nested into the global model ECHAM5 [35]. In contrast to their driving global models, regional climate models integrate topography and can project climate change at a much higher spatial resolution which enhances the quality of studies on climate change impacts [36]. Our future projections refer to the IPCC A1B and B1 emission scenarios for greenhouse gases [37]. In short, the A1B scenario is characterised by a rapid economic growth in an integrated world with a balanced technological emphasis on fossil and non-fossil energy sources. The B1 scenario is based on the same economic growth as in A1B but with a more rapid change towards a service and information economy. Climatic data were averaged over time periods 2011–2040, 2041–2070 and 2071–2100 for each scenario separately. Bioclimatic variables for modelling future climate projections were calculated in the same way as they are provided by http://www.worldclim.org [32] for current conditions.

Non-analogue climatic conditions are a problematic issue in projections [38]. We used a Multivariate Environmental Similarity Surface (MESS) analysis introduced by Elith et al. [39] to detect regions where projections are inappropriate due to dissimilarity in values of the used variables for training and projecting the model [40]. The MESS-analysis measures the similarity between those environments used to train the model and the new projected environments for any grid cell [39], [40]. Occurrence records and climatic data were prepared in ArcGIS 10.0, correlation analysis was performed in PASW Statistics 18, Jackknife test to measure the variable's importance is implemented in MaxEnt 3.3.3e.

#### Model runs

All models were generated using the maximum entropy algorithm. Maximum entropy basically is a machine-learning technique combining species occurrence data with detailed climatic and environmental datasets [41], [42]. This algorithm implemented in MaxEnt software computes a probability distribution covering the study area that satisfies a set of constraints which are derived from environmental conditions at species presence records. The algorithm then chooses a distribution with maximum entropy within all possible distributions [41]. MaxEnt generally performs better than other presence-only or pseudo-presence-only models [41], [43], [44], which becomes especially apparent by using small numbers of species occurrence records [45]–[47]. Furthermore, the influence of spatial errors in species occurrences on model performance of MaxEnt due to e.g. inaccurate georeferences is less severe in comparison to other algorithms [48].

The MaxEnt-models settings have been adapted from a previous study concerning the projections of climatic suitability for *Aedes albopictus* in Europe [49]. We randomly selected 70% of the occurrence data to train each model and used the remainder to test each model in order to evaluate model accuracy (e.g. [50]). Models were replicated 100 times for each species and the results were finally averaged. We used both, threshold-dependent as well as threshold-independent quality criteria. Eleven binary omission rates were calculated as the proportion of test respective training points that were not predicted at a threshold probability that equalled the minimum probability on any pixel containing an occurrence point [42]. Those were tested using one-sided p-values for the null hypothesis that test points are predicted no better than by a random prediction with the same fractional predicted area. This was practiced previously for the evaluation of model results for *Lutzomyia spp.* [24]. Furthermore, model performance was evaluated using area under the receiver operator curve (AUC) statistics, which compares how likely a random presence site will have a higher predicted value in the model than a random absence [43]. The receiver operator curves appeals to be independent on a user-defined threshold for determining presence versus absence. We limited the study area to the geographic extent of the sampling distribution (see chapter Species presence records and climatic variables) in order to avoid inflated AUC-scores that are associated with geographical extents that go beyond the presence environmental domain [51], [52]. All models were built in the latest available version (MaxEnt 3.3.3e).

### Least-cost path for species dispersal

It is well known that species dispersal ability is dependent on the environment and varies strongly with landscape structure [53]. In order to make projections more realistic according to spatial characteristics, we used a least-cost path analysis based on graph theory [54]–[56] to determine the most likely way for *Phlebotomus spp.* to move across a spatio-temporally changing landscape. The path function indicates the least efforts (“costs”) for a species in moving through any particular cell in the respective landscape [57]–[59]. Least-cost path analyses are frequently used to determine potential dispersal pathways for mammals [60]–[63] but have also been applied to insects [58].

#### Definition of cost surfaces and calculation of distances and backlinks

Our aim was to identify the species-specific least-cost pathway for potential movement of the five *Phlebotomus spp.* to each of the modelled climatically suitable habitats during the 21^st^ century for the selected time-periods and for each scenario, respectively. Therefore, we created three different species-specific cost surfaces. The first cost-surface was generated for costs arising for the species movement to climatically suitable habitats of the upcoming time-period (2011–2040). The second one was built for the movement up to mid-century (2041–2070) and finally to the end of the century (2071–2100). The respective cost factors are listed in Table 2. Each cost surface includes as well temporarily stable as varying environmental landscape features.

**Table 2:** Cost factors within the defined cost surfaces for *Phlebotomus* species.

Landscape feature	Value or area	Cost factor
Elevation	0–800	0
	801–1200	1
	>1200	2
Further landscape features	River valleys	0
	Non-valleys	4
	Sea	-
Climatic suitability (taken from MaxEnt-models, averaged over two subsequent time-periods)	0.81–1.0	0
	0.61–0.8	1
	0.41–0.6	2
	0.21–0.4	3
	0.01–0.2	4
Wind speed one metre above the ground(in m/s, averaged over two subsequent time-periods)	0.01–1.5	0
	1.51–2.5	1
	2.51–3.5	2
	>3.5	3

Factors are surfaces were generated by considering both, spatio-temporal stable and variable environmental conditions within the 21^st^ century. Two cost factors are stable within the 21^st^ century: River valleys are considered as the preferred breeding sites for sandflies [2]. Hence, regions which include river valleys are attributed with costs. Sea as absolute barrier cannot be crossed. Beyond, an altitudinal cost structure was developed in accordance to the preferred elevation of sandflies [2]. Two factors vary in the 21^st^ century: MaxEnt-values of climatic suitability range theoretically from 0 (unfavourable conditions) to 1 (perfect conditions). These values are classified and attributed with costs in accordance to the suitability; the lower the suitability the higher the costs. This factor is species-specific. Cost factors for wind speeds are related to the observations concerning wind-speed dependent flight activity. They are taken from findings of Lane [94] concerning the highest flight activity up to 1.5 m/s; from Quate [95] who observed a reduced flight activity between 1.5–2.5 m/s and Roberts [96] who noticed no flight activity above wind speeds of 3.5 m/s. Climatic suitability and wind speed were averaged over two subsequent time-periods.

**Figure 1:**
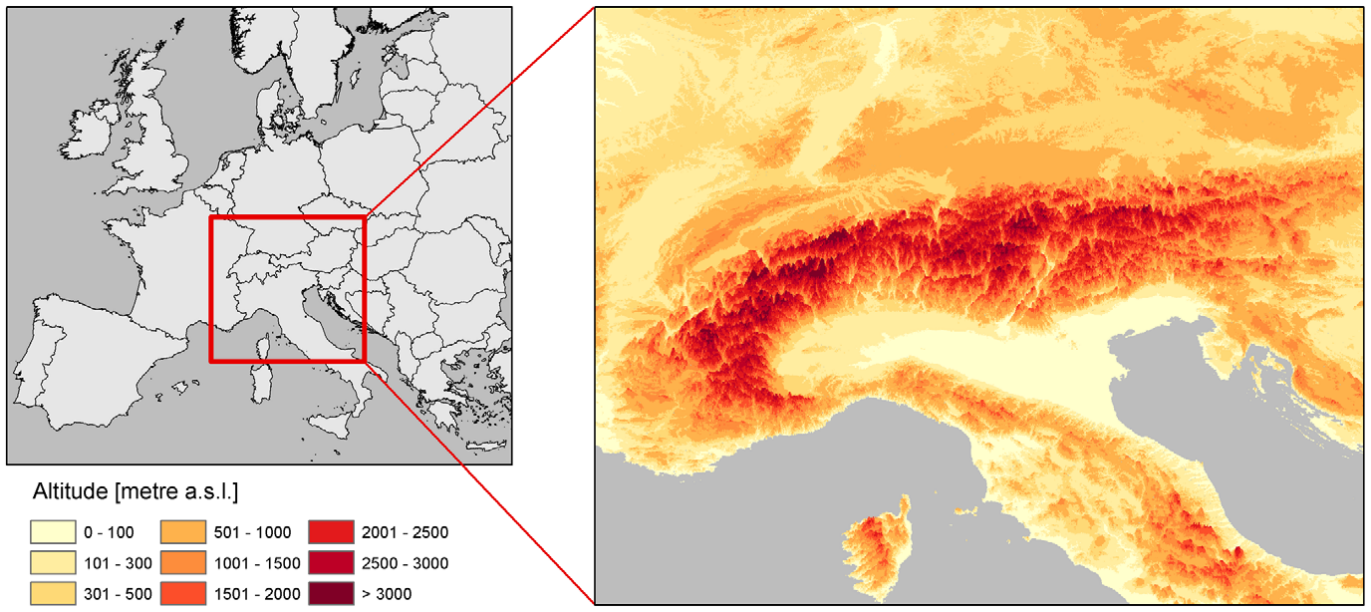
Location and topography of the Alps. Elevation is visualised in tenfold vertical exaggeration. The Alps are the highest mountain range in the continental interior of Europe separating the Mediterranean region from Central Europe and extending from France (West) through Switzerland and Italy (South) into Austria, Germany (North) Slovenia, and Croatia (East). Alpine regions are considered as the main natural barrier for natural sandfly dispersal.

a) Environmental landscape features that do not to change in the 21^st^ century were consequently considered as constant (stable) cost factors in all created cost surfaces: River valleys provide the preferred breeding sites due to high temperatures and moist and humid soils [64], [65] and can hence be considered as preferred dispersal corridors. We buffered this features with a distance of ten kilometres for consistency with the climatic data and attributed it without costs. Only the surrounding landscape was addressed with costs. Additionally, increasing elevation was attributed with rising costs [2]. The topographical structure of Europe is presented in Figure 1. Sea surfaces and high mountains were considered as “absolute barriers” which cannot be crossed. These costs were the same in all cost surfaces throughout the 21^st^ century and for all species.

b) Within the cost surfaces temporally varying environmental landscape features in the 21^st^ century (due to climatic effects) were integrated: The species-specific changing climatic suitability of an area between two subsequent time-steps (current-2011/2040, 2011/2040–2041/2070, 2041/2070–2071/2100) was integrated as a further cost factor in the respective cost surfaces. For this purpose the projected climatic suitability was averaged over two subsequent time-periods. The values of climatic suitability were taken from MaxEnt-models. Regions that have to be overcome between two time-periods but would persist to remain outside of the preferred bioclimatic niche were attributed with higher costs. We furthermore integrated wind speed in the cost surface as sandflies react very sensitive to high wind speed by reducing flight activity [17], [21]. Data of current and projected wind speed (for A1B and B1 scenario) are taken from CCLM. Data were averaged for the equivalent time-periods, which were already used to model climatic suitability for the single sandfly species (2011–2040, 2041–2070 and 2071–2100). It is realised that sandflies predominantly prefer to be active near the soil surface (up to one metre above the ground) and usually do not exceed two metres above the ground [64]. The provided data of wind speed, however, are given for a height of ten metres above the ground. In consequence, we applied the wind profile power law that is derived from the logarithmic wind profile equation for the lower atmosphere in order to relate wind speeds given at one height to another [66]. The equation to calculate wind speed in one metre was calculated for each time-period by:
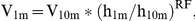
with V_1m_ representing wind speed in one metre, V_10m_ representing the (given) wind speed in ten metre above the ground, h_1m_/h_10m_ = 0.1 (height of one metre, divided by the height of ten metre - where the velocity is given by data of CCLM) and RF (roughness factor). Land cover decreases the near surface wind speed due to the roughness of the landscape features. The RF should not be considered as spatially constant as it varies for different surface obstacles, which must be taken into account [67]. Therefore, we integrated land cover data provided by http://earth.esa.int [68] for the calculation of the near-surface wind speed. In our study, land cover is considered not to change during the 21^st^ century. We reclassified the provided map of land cover in Europe into three classes (with different RF) proposed by Kleemann and Meliss [69]:

1. Low RF ( = 0.16): cropland, grassland vegetation, bare areas
2. Mid RF ( = 0.28): different types of forests, vegetation dominated by trees and rural communities
3. High RF ( = 0.40): urban areas

Then, the wind speeds were averaged over two subsequent time-periods of species movement as it was done for the modelled climatic suitability. For all species, the development in wind speed was attributed with equal costs (Table 2). Due to these temporal changing factors (species climatic suitability and wind speed) different cost surfaces were generated.

#### Determining the least-cost path based on the generated cost surfaces

Determining the least-cost path requires cost distance and cost backlink calculations as inputs which are both assigned on the basis of the defined cost surfaces. Cost distances were calculated in order to account for the minimal accumulated travel costs that accrue by travelling with increasing distance from the source to the target area [63]. The cost backlinks indicate the direction for each grid cell to which direction the costs are cheapest. Details on calculations of cost distance and assignment of cost backlink can be found elsewhere (for review see [70]). Our initial source grid for species occurrences as starting points included all areas with documented current European presence records. As destination area we defined Central Europe (Austria, Germany and Switzerland) for all species throughout the 21^st^ century. The natural dispersal ability of sandflies is limited. Generally, the flight range around their breeding area is about one kilometre [71]. The flight range for *P. ariasi*, however, can reach two kilometres [17]. In Mediterranean areas, sandflies are able to establish up to three generation each year [2]. Therefore, we limited the maximal natural range expansion for *P. ariasi* to six kilometres per year (180 km/30 year time interval) and for the other sandfly species to three kilometres per year (90 km/30 year time interval) for moving through any particular cost surface.

**Figure 2:**
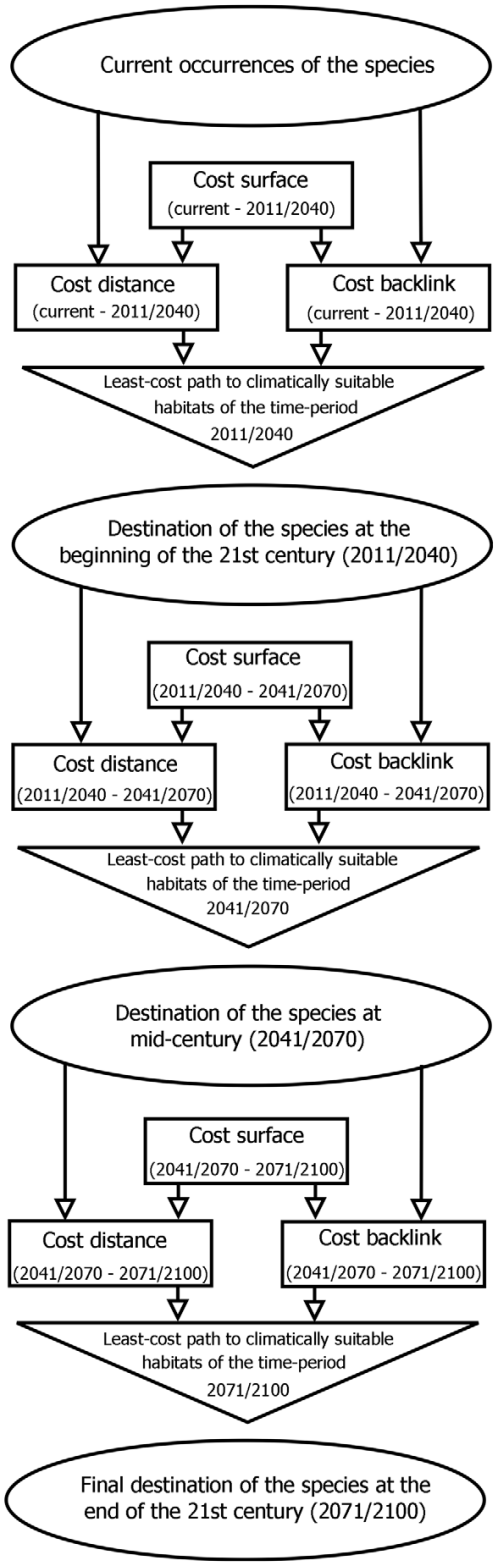
Principle of the least-cost analysis. Least-cost analysis was used to determine the most likely way of natural dispersal sandfly species in the 21^st^ century.

In our study, species are assumed to be able to establish in climatically suitable habitats indicated by values higher than 0.5 of the MaxEnt-models. This typically corresponds to values of the climatic suitability on the respective presence records [42]. Therefore, those regions were indicated as species occurrences of the subsequent time period (2011–2040) that overtop the threshold of suitability and that are connected via the initial least-cost path. Those areas were defined as new starting points for the least-cost path to the expected climatically suitable habitats at mid-century (2041–2070). This procedure was repeated for a third time in order to determine reachable location of the sandfly species at the end of the 21^st^ century (2071–2100). The principle is illustrated in Figure 2. Least-cost analysis was performed using distance functions within the “Spatial Analyst Tool” implemented in ArcGIS 10.0.

## Results

### Model results for species climatic suitability

AUC-values yielded in high scores for five species (Table 1). Binominal tests indicated that tests points are predicted better by the model than a random prediction with the same factional predicted area at the significance level p<0.01. Similarity between current and projected climate is analysed by MESS-analysis. Highest similarity in projections is indicated for southern parts of Germany, the northernmost regions of Switzerland as well as for eastern and north-eastern parts of Austria. Instead, lowest similarity exists for alpine regions and northern Germany. However, our projections seem not to be biased by non-analogue climate.

**Figure 3:**
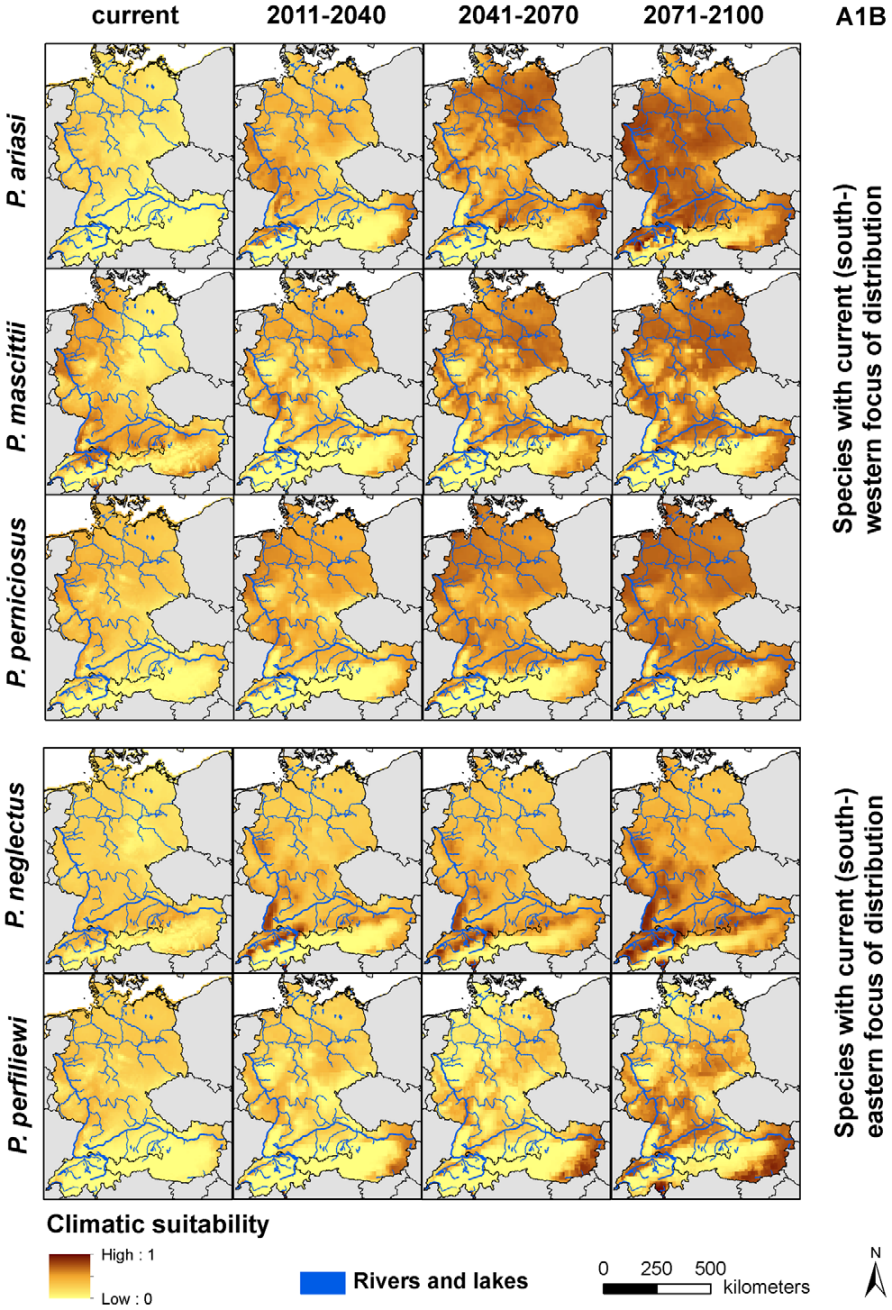
Current and projected climatic suitability for five *Phlebotomus* species. Values of climatic suitability range theoretically from 0 (unfavourable conditions) to 1 (perfect conditions). Projections refer to the A1B scenario.

In general, climatic suitability can be expected to increase for all species in the 21^st^ century (Figure 3 and S1, Table 3 and S1). This is in accordance with the first part of our first hypothesis assuming increasing climatic suitability for the species in Central Europe. Nevertheless, we cannot completely verify the second part of our first hypothesis of more favourable conditions in the (south-) westernmost parts of Central Europe for species with current (south-) western focus of European distribution and the opposite for species which are currently distributed in (south-) eastern parts of Europe. Overall, projections based on the A1B scenario (Table 3) represent higher suitability for species in comparison to projection of the B1 scenario (Table S1). Nevertheless, the spatial patterns of potential climatically suitable habitats remain to be the same for both scenarios. The detailed annotation of climatic suitability in the following refers to the A1B scenario.

**Table 3:** Current and projected climatic suitability for *Phlebotomus* species in Central Europe.

	Central Europe				Austria				Germany				Switzerland			
	current	2011–2040	2041–2070	2071–2100	current	2011–2040	2041–2070	2071–2100	current	2011–2040	2041–2070	2071–2100	current	2011–2040	2041–2070	2071–2100
*P. ariasi*	0.14 (+/_−_0.10)	0.32 (+/_−_0.13)	0.38 (+/_−_0.17)	0.54 (+/_−_0.17)	0.02 (+/_−_0.02)	0.17 (+/_−_0.22)	0.29 (+/_−_0.19)	0.39 (+/_−_0.21)	0.17 (+/_−_0.08)	0.36 (+/_−_0.11)	0.43 (+/_−_0.11)	0.60 (+/_−_0.10)	0.07 (+/_−_0.08)	0.18 (+/_−_0.24)	0.11 (+/_−_0.15)	0.39 (+/_−_0.29)
*P. mascittii*	0.28 (+/_−_0.19)	0.30 (+/_−_0.20)	0.40 (+/_−_0.23)	0.47 (+/_−_0.25)	0.26 (+/_−_0.17)	0.22 (+/_−_0.19)	0.28 (+/_−_0.24)	0.28 (+/_−_0.23)	0.32 (+/_−_0.18)	0.36 (+/_−_0.16)	0.48 (+/_−_0.18)	0.55 (+/_−_0.18)	0.21 (+/_−_0.18)	0.12 (+/_−_0.14)	0.14 (+/_−_0.16)	0.15 (+/_−_0.19)
*P. perniciosus*	0.25 (+/_−_0.14)	0.35 (+/_−_0.19)	0.46 (+/_−_0.20)	0.52 (+/_−_0.22)	0.09 (+/_−_0.10)	0.17 (+/_−_0.20)	0.26 (+/_−_0.23)	0.33 (+/_−_0.26)	0.31 (+/_−_0.10)	0.42 (+/_−_0.14)	0.53 (+/_−_0.12)	0.60 (+/_−_0.12)	0.13 (+/_−_0.15)	0.10 (+/_−_0.17)	0.15 (+/_−_0.23)	0.19 (+/_−_0.26)
*P. neglectus*	0.23 (+/_−_0.10)	0.31 (+/_−_0.15)	0.38 (+/_−_0.14)	0.49 (+/_−_0.16)	0.18 (+/_−_0.13)	0.23 (+/_−_0.20)	0.36 (+/_−_0.20)	0.46 (+/_−_0.20)	0.24 (+/_−_0.08)	0.34 (+/_−_0.11)	0.38 (+/_−_0.09)	0.49 (+/_−_0.12)	0.23 (+/_−_0.17)	0.30 (+/_−_0.30)	0.40 (+/_−_0.28)	0.53 (+/_−_0.31)
*P. perfiliewi*	0.10 (+/_−_0.06)	0.10 (+/_−_0.13)	0.19 (+/_−_0.17)	0.33 (+/_−_0.23)	0.04 (+/_−_0.03)	0.22 (+/_−_0.22)	0.28 (+/_−_0.28)	0.36 (+/_−_0.32)	0.12 (+/_−_0.05)	0.07 (+/_−_0.06)	0.17 (+/_−_0.12)	0.34 (+/_−_0.20)	0.05 (+/_−_0.04)	0.17 (+/_−_0.26)	0.17 (+/_−_0.20)	0.29 (+/_−_0.30)

Noted are mean values and standard deviation in brackets. Projections refer to the A1B scenario.

#### Climatic suitability for species with current (south-) western focus of distribution

Results for species with current (south-) western focus of distribution (*P.ariasi*, *P. mascittii* and *P. perniciosus*) show - regardless of slight differences - a comparable tendency in spatial patterns of projected climatic suitability for the upcoming time-period (Figure 3 and S1). Expectedly, these species achieve highest values of current and projected climatic suitability in the westernmost parts of Germany and Switzerland. Projections for the conditions from mid-century onwards, however, indicate increasing suitability for the eastern parts of the countries. Interestingly, moderate suitability is indicated for *P. perniciosus* on western parts of Germany and the coast of the North Sea for the current climatic conditions. In those regions no presence of the species is documented up to now. Favourable conditions for *P. mascittii* and *P. perniciosus* can be expected in north-eastern Germany at the end of the century and in less extent also for *P. ariasi*. Instead, *P. ariasi* will achieve highest values of climatic suitability in Switzerland. Nevertheless it is worth mentioning that favourable conditions can be expected for all species in certain river valleys in the northern and north-eastern parts of Switzerland on the border to France and Germany and along the “Danube valley”. This becomes especially apparent regarding the projections for the end of the 21^st^ century. For *P. mascittii* climatic suitability will persist in eastern (including south-eastern and north-eastern parts) of Austria. For *P. ariasi* and *P. perniciosus* suitability can be expected to increase in those regions. Austrian alpine regions remain to persist outside the preferred niche of all three species throughout the 21^st^ century.

#### Climatic suitability for species with current (south-) eastern focus of distribution

The results of the current and projected climatic suitability for the two species with (south-) eastern European focus of distribution (*P. neglectus* and *P. perfiliewi*) differ remarkably. For *P. neglectus*, habitats in Upper Austria along the the “Lake Constance” regions, located in the southern parts of Germany and northern parts of Switzerland, are detected to be adequate under current conditions. For the upcoming time-period, it can be expected that especially the “Upper Rhine Valley” in the southwest of Germany will provide suitable climatic conditions. Starting at mid-century, almost all regions in Germany will provide favourable conditions for species establishment. At the end of the century, additionally, northern and southern parts of Switzerland will achieve favourable climatic conditions. Then, only the highest Alpine regions are expected to remain climatically unsuitable the establishment of *P. neglectus*. The current and projected values of climatic suitability for *P. perfiliewi* differ spatially from those that are calculated for *P. neglectus*. Currently, climatic requirements of *P. perfiliewi* will not be fulfilled in Central Europe. Favourable conditions can be expected for the up-coming time period and mid-century higher in spatially limited areas for southernmost parts of Switzerland - canton Ticino - and (south-) eastern parts of Austria. Germany remains unfavourable for the establishment of *P. perfiliewi* until mid-century. At the end of the 21^st^ century, the river valley of the “Rhine” and “Danube” will provide preferable climatic conditions for *P. perfiliewi*.

### Least-cost path analysis

**Figure 4:**
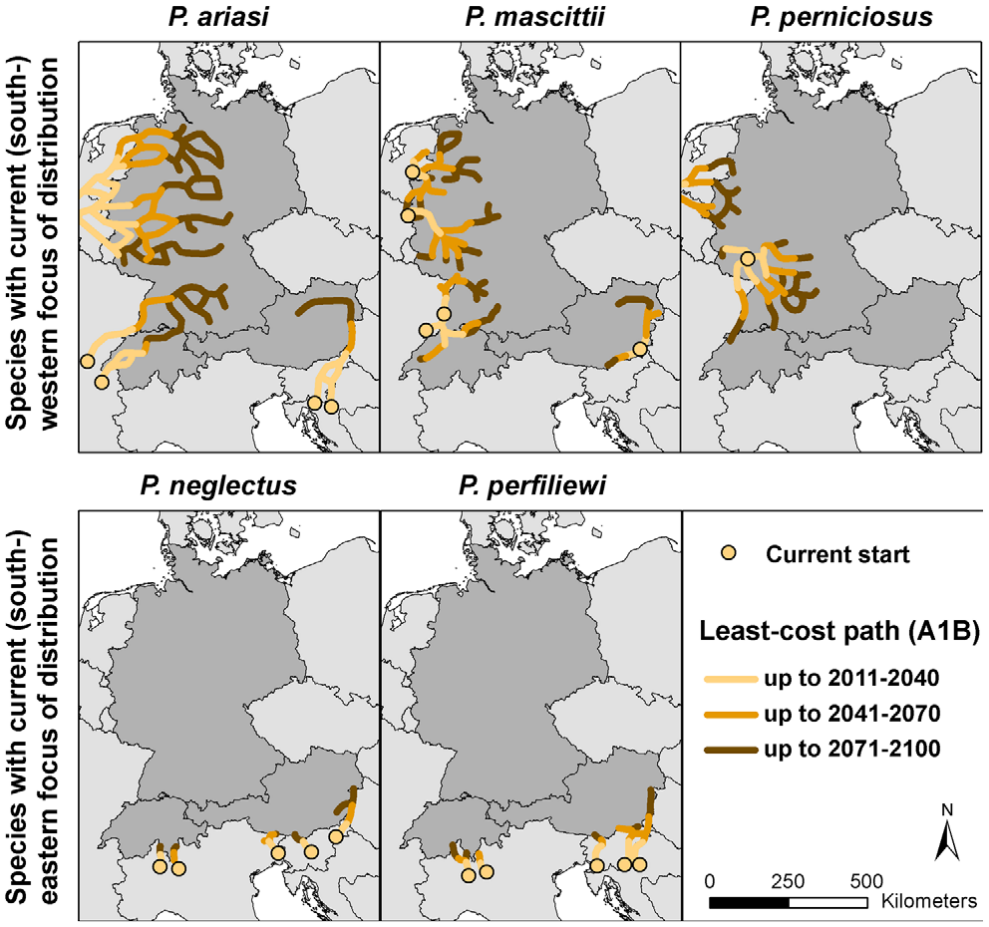
Least-cost paths for *Phlebotomus* species. The detected pathways indicate direction of spread in the 21^st^ century. Spatio-temporal varying climatic suitability and wind speed included in the cost surface that must be crossed by species in the 21^st^ century refer to the A1B scenario.

In general, projections hint on spreading tendencies for all studied *Phlebotomus spp.* to areas where they have not occurred so far in both scenarios (Figure 4 and S2). Nevertheless, sandfly species will not be able to become established in all climatically suitable areas of Central Europe according to the limited natural dispersal ability. The detected dispersal pathways show some differences between the two applied scenarios, in contrast to the modelled climatic suitability, where just temporal but no spatial variations are pointed out.

The first part of our second hypothesis that species with current (south-) western focus of distribution are likely to disperse eastwards can be affirmed only for *P. perniciosus* but not for *P. ariasi* and for *P. mascittii*. We cannot confirm the part of the second hypothesis that the Alps will prohibit completely a northward spread for the species which are currently distributed in (south-) eastern European regions. However, it is very likely that the Alps will decelerate the range expansion.

#### Least-cost path for species with current (south-) western focus of distribution

In contrast to the similar tendency in climatically suitable habitats, dispersal pathways for species with current (south-) western focus of distribution (*P. ariasi*, *P. mascittii* and *P. perniciosus*) will differ. *P. ariasi* is characterised by the highest dispersal ability and seems to spread to Switzerland and Germany from recent occurrences in France. Consequently, the western parts of these countries can be expected to be occupied already in the upcoming time-period. From then on, the species seems to spread along the border of Germany and Switzerland to Bavaria in the southeast of Germany until the end of the century. Additionally, *P. ariasi* will spread to north-eastern parts of Germany until the end of the century in A1B but not in the B1 scenario. A further dispersal pathway is detected starting from Croatian and Slovenian occurrences directed to the (south-) eastern parts of Austria. Up to the end of the century, *P. ariasi* will also be able to occupy eastern and north-eastern parts of Austria. Two pathways are detected for *P. mascittii* starting from the “Upper Rhine Valley” in the southwest of Germany, which are either directed northwards or southwards (to the northeast of Switzerland). Additionally, a direct spread from France to Switzerland is identified. As two pathways are directed to northern parts of Switzerland, this region seems to be especially endangered regarding species establishment, expectedly from mid-century onwards. The north-eastern expansion for *P. mascittii* in Germany is solely indicated when applying the A1B scenario. Additionally, two pathways are detected from “Carinthia” in Austria. A northward spread to the river valley of the “Danube” and a westward spread along the Slovenian and Italian boarder can be expected in both scenarios. From the recent occurrences of *P. perniciosus* in the southwest of Germany, potential pathways are determined mainly to southern and eastern directions. Along the southernmost parts of Germany *P. perniciosus* will disperse to the southeast of Germany (Bavaria) until the end of the 21^st^ century. Beyond that, western parts of Germany will be reached from an expected movement from French regions across Belgium. This range expansion is more pronounced when applying the A1B scenario. Switzerland and Austria seem not to be exposed to a direct northward spread from the species across the Alps.

#### Least-cost path for species with current (south-) eastern focus of distribution

In comparison to species with current (south-) western focus of distribution, potential range expansions for *Phlebotomus spp.* with current (south-) eastern focus of distribution (*P. neglectus* and *P. perfiliewi*) are more restricted. The detected least-cost paths for *P. neglectus* are rather diverse. For this species, several pathways indicate a northward spread to locations in Switzerland (from Italy) and Austria (from Slovenia). Expectedly at mid-century, the southern parts of Switzerland and Austria will be achieved by the specimen. Only in A1B scenario, *P. neglectus* is expected to be able to spread to eastern regions of Austria and establish permanent populations there. There is no evidence that this species may move further northwards to Germany. *P. perfiliewi* is currently established in northern Italy. This species will reach southern areas of Switzerland and Austria within the period 2011–2040. Slight range expansions to eastern Austria can be expected until mid-century, but these will become more pronounced towards the end of the century. Further dispersal of *P. perfiliewi* in Switzerland is unlikely to happen. Germany seems not to become a part of this species' range during this century.

## Discussion

### Relevance and generality of the study

Our aim was to determine future occurrences of five *Phlebotomus spp.* with spreading tendencies in the face of a changing climate. These sandflies serve as proven or assumed (*P. mascittii*) vectors of *Leishmania infantum* causing the leishmaniasis. Knowledge concerning the potential future presence of disease vectors is a first step towards an accurate and efficient risk assessment of vector-borne diseases [72]. Conventional static bioclimatic niche modelling can be extended by novel avenues for instance regarding species-specific abilities to disperse [73]. Therefore, we integrated species-specific dispersal pathways to the detected climatically suitable habitats. Within this study, we focus on active and natural dispersal of the species and excluded potential human assistance for range expansions, for instance via the transport of subtropical plants containing eggs or larvae in the moist substrate. However, these effects are not clearly understood and hence not included in this analysis. The results of this study represent the minimum range expansion of *Phlebotomus spp.* that is only related to active and natural movement in a changing environment without potential human-assistance.

Our results suggest that the development of Central European climate will increasingly support suitable habitats for phlebotomine sandflies. This general trend will become even more pronounced in the second half of the 21^st^ century. We project sandfly establishment in formerly non-endemic areas. This will additionally increase the risk of emerging sandfly-borne diseases in Central Europe such as leishmaniasis. Nevertheless, it is unlikely that sandflies will reach and occupy the provided climatically suitable habitats entirely. During the upcoming years, a spatial focus of surveillance regarding potential new-establishment of *Phlebotomus spp*. with current south-(western) focus of distribution should be directed for western parts of the German state “North Rhine-Westphalia”. These species may additionally occupy the regions around the “Lake Constance” (southern part of Germany and northern part of Switzerland). Furthermore, Switzerland must be aware on potential sandfly occurrences around the river valley of the “Aare” and the “Lac dé Neuchâtel’ already within some years. In Austria, especially the south-eastern states “Carinthia’, “Styria’ and “Burgenland’ seem to be at risk by new-infestations due to the spreading tendencies of *P. mascittii* and *P. ariasi*. In the case of sandfly species with current south (-eastern) focus of distribution (*P. neglectus* and *P. perfiliewi*), the canton “Ticino” in southern Switzerland must be alert. According to our results, especially the regions around “Lago Maggiore” and “Lago di Lugano” should be monitored systematically. Furthermore, the Austrian regions neighbouring Slovenia and Hungary should be prepared for the establishment of these sandfly species in the near future.

### Limitations

The main limitation in our projections refers to the accuracy of georeferencing maps of sandfly distribution. We have chosen an algorithm that is capable to cope with this source of uncertainty [48]. Expectedly, improved model projections would arise with geographically more accurate point data. However, the intension of this paper is rather to provide a methodological approach. Recently, it has been pointed out that pixel values of predictor variables in close proximity will be highly correlated, which would reduce the effect of inaccuracy in spatial data set of species occurrences [74]. Furthermore, when comparing the reports for cases of autochthonous leishmaniasis in regions that were considered as being non-endemic (e.g. [9]) with documented presence records of sandflies leads to the assumption that sandflies may be wider distributed than realised. However, as it is unknown which species acted in such regions as vectors, only documented presence records at the species level of the *Phlebotomus spp.* can reasonably be integrated.

Assuming climate is generally considered to be suitable for the permanent establishment of populations, the presence of *Phlebotomus spp.* is additionally dependent on land cover e.g. forest, agriculture and urban areas [75], [76]. In this study, we integrated altitudinal structures such as river valleys and mountain ridges in least-cost path analysis. In order to recalculate wind speed we additionally integrated land cover data as surface roughness to decelerate near-surface wind speed. Climate change may contribute to alterations in land use and cover, due to warming, changes of precipitation regimes and increases of climatic extreme events such as droughts or floods. This is likely to affect the spatial structure of agricultural systems. In addition to direct climatic impacts, these changes of land cover will additionally affect the spread and distribution of sandflies. However, land cover and land use changes depend on complex processes of decision making under specific political and economical conditions [77] and are hence difficult to project. Therefore land use and cover were considered to remain constant in this study. In general, biotic interactions such as predation or competition are crucial for species distribution [78]. The modifications of the ecological links or networks of an organism by climate change can substantially alter the realised niche of species population [79]. In Germany, *P. perniciosus* and *P. mascittii* do not co-exist at the same locations [29]. This can be a result of diverging invasion pathways or of competitive exclusion in the respective regions. Unfortunately, knowledge on biotic interactions of sandflies is scarce. Furthermore, one has to bear in mind that presence of phlebotomine sandflies is dependent on humans and their social factors, for instance living conditions [80], [81]. However, all these factors become more important for species distribution on smaller spatial scales than applied in this study [82], [83].

Concerning the least-cost analysis for species movement it has to be noted that the attributed costs are based on assumptions and/or preliminary observations and hence may not include all of the relevant factors [55], [61]. For instance, it is questionable whether humans assist in the spread of sandflies. Nevertheless, in comparison to mosquitoes, direct human effects on dispersal are of minor importance. Furthermore, the species movement behaviour must not necessarily be optimal or well adapted in human-modified landscapes [53]. Especially dispersal behaviour of individuals between populations may differ from the general tendency at the metapopulation level [58].

Besides the effects of changes in long-term climatic conditions used in this study, extreme weather events are expected to increase in Europe [84]. This will influence organisms and ecosystems remarkably [85], [86]. It has been shown that climatic variability in general [87] and extreme weather events such as floods particularly affect sandfly occurrences [88]. In order to integrate weather extremes in a satisfactory quality within climatic projections, a further downscaling of their spatial resolution to the local scale is required [89]. This is the only way to account for the contribution of weather extremes on disease vectors in risk analysis.

### Strengths

Bioclimatic envelope models are powerful tools to envisage potential responses in species distribution to climate change from regional to global scales [82], [90]. They can be seen as a useful first filter for approximations of the impact of climate change on the species distribution [82], [91]. A well-adapted modelling approach is required to project climate change effects on species [44]. Therefore, we selected MaxEnt as algorithm, due to better performances in comparison with further presence-only and (pseudo-) presence-only algorithms (see chapter Model runs for details). Results yielded in high model quality criteria, emphasised by threshold-dependent and independent criteria for *Phlebotomus spp*.

In order to cope with the general uncertainty in species distribution modelling regarding the climatic evolvement [78], we projected the climatic suitability based on two IPCC [37] scenarios (A1B and B1) that best illustrate the respective storyline. We choose bioclimatic variables that are considered to be biologically meaningful variables for model input. Our projections of future climatic suitability refer to data of the European regional climate model CCLM, which is nested into the well-established global climate model ECHAM5 [35]. In comparison to their driving global models, regional patterns of climate change are projected more precisely, which enhances the quality of climate impact studies [36]. For instance, global climate models fail particularly in replication of observed wind speeds. Obviously, projections of changes in wind speed profit from downscaling to the regional level [92]. Furthermore, potential regions with non-analogue climatic conditions and where hence projections are inappropriate were excluded via MESS-analysis.

Evidently, the consideration of the dispersal capacity of insects in a changing climate improves the quality of projections of species distribution [93]. Hence, we combined projected climatic suitability in the 21^st^ century with dispersal ability for five *Phlebotomus spp*. We practiced least-cost analysis for future movement patterns by including temporarily stable (elevation, landscape features) and variable factors (wind speed and development of climatic suitability). This allows integrating and combining expert knowledge on sandfly ecology and biology with statistical methods. In doing so, potential species-specific dispersal pathways can be pointed out. This offers the opportunity to distinguish between climatically suitable habitats that can be reached by invasive species and those that are not accessible to them.

The proposed method for the detection of dispersal pathways can be applied to other invasive and mobile disease vectors in the face of climate change. For this purpose, however, species-specific cost surfaces have to be generated. The Alps, for instance, may not be seen as crucial natural barrier for the mainly human-assisted spread of invasive mosquitoes such as the Asian tiger mosquito (*Aedes albopictus*). However, they are an efficient natural barrier for other species.

### Conclusions

Here, we provide a powerful methodological approach to extend conventional bioclimatic envelope modelling of disease vectors by species specific dispersal ability. Our findings promise more realistic projections concerning the vector species future distributions. We identify those Central European regions that are especially exposed to the emerging threat of disease vectors in the light climate change. For the modelling of hitherto neglected vector-connected risks, expertise from various scientific disciplines has been taken into account. Proactive monitoring activities and development of feasible adaptation strategies are required before the establishment of disease vectors may take place. Hence, those analyses help to focus control programmes on specific areas at risk on a regional scale. Due to the identification of spatial hot-spots for such activities, cost- and time-efficient surveillance strategies can be developed. This enables target-orientated counteractions directed against the suggested spread of disease vectors in time. Consequently, the risk of disease transmission in formerly non-endemic areas can be reduced. Once disease vectors such as sandflies are established, vector control and disease prevention have proven to be limited.

## Supporting Information

Figure S1 Current and projected climatic suitability for five *Phlebotomus* species. Values of climatic suitability range theoretically from 0 (unfavourable conditions) to 1 (perfect conditions). Projections refer to the B1 scenario.
(TIF)

Figure S2 Least-cost paths for *Phlebotomus* species. The detected pathways indicate direction of spread in the 21^st^ century. Spatio-temporal varying climatic suitability and wind speed included in the cost surface that must be crossed by species in the 21^st^ century refer to the B1 scenario.
(TIF)

Table S1 Current and projected climatic suitability for *Phlebotomus* species in Central Europe. Noted are mean values and standard deviation in brackets. Projections refer to the B1 scenario.
(DOC)
