# Supplementary figures and images for: Combining Climatic Projections and Dispersal Ability: A Method for Estimating the Responses of Sandfly Vector Species to Climate Change
Source: PLoS Negl Trop Dis. 2011 Nov 29;5(11):e1407. doi: 10.1371/journal.pntd.0001407 (PMC3226457; doi:10.1371/journal.pntd.0001407)

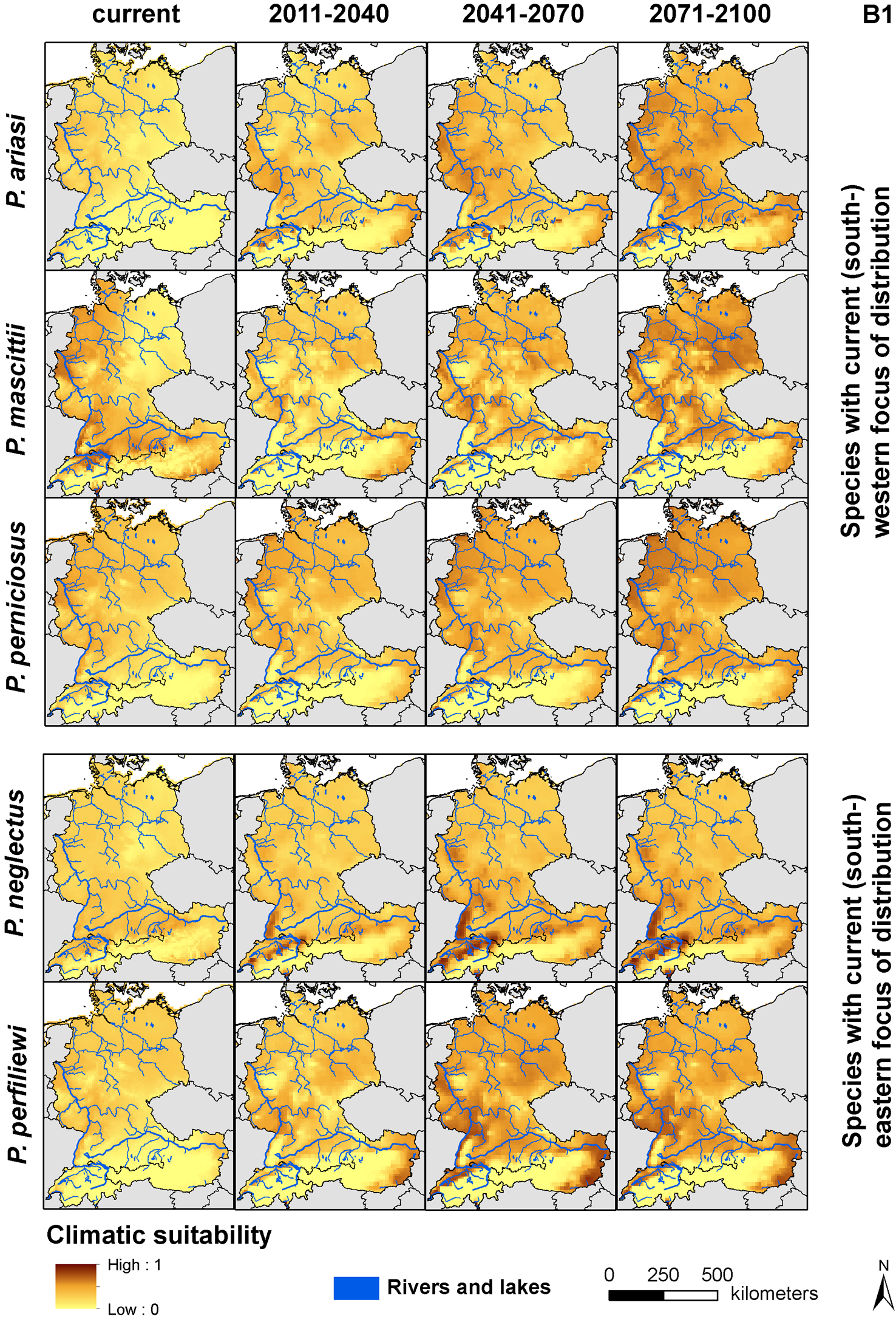

Supplement: Figure S1 — Current and projected climatic suitability for five Phlebotomus species. Values of climatic suitability range theoretically from 0 (unfavourable conditions) to 1 (perfect conditions). Projections refer to the B1 scenario. (TIF) [file pntd.0001407.s001.tif]

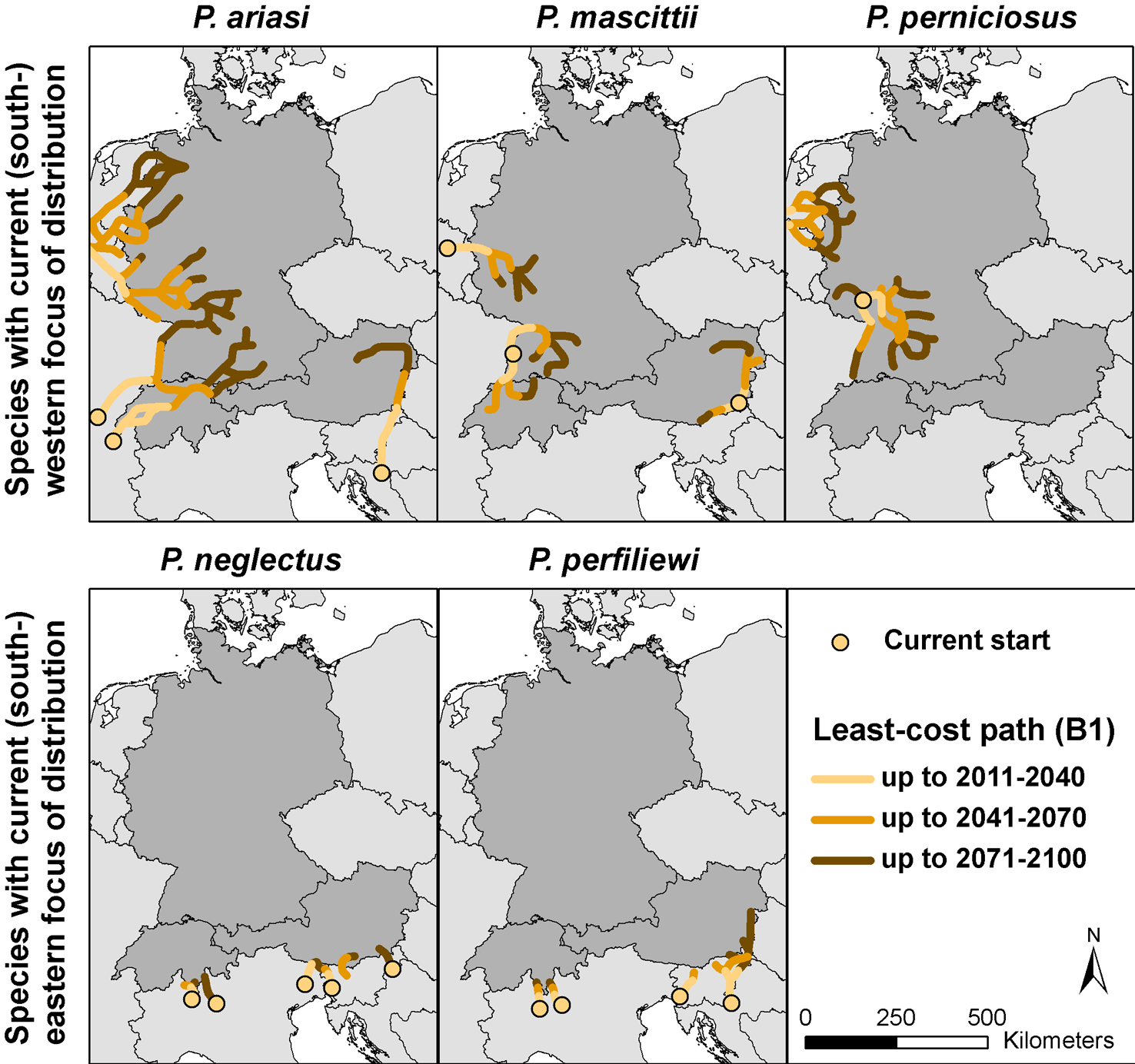

Supplement: Figure S2 — Least-cost paths for Phlebotomus species. The detected pathways indicate direction of spread in the 21st century. Spatio-temporal varying climatic suitability and wind speed included in the cost surface that must be crossed by species in the 21st century refer to the B1 scenario. (TIF) [file pntd.0001407.s002.tif]
