# Supplementary material for: Combining Climatic Projections and Dispersal Ability: A Method for Estimating the Responses of Sandfly Vector Species to Climate Change
Source: PLoS Negl Trop Dis. 2011 Nov 29;5(11):e1407. doi: 10.1371/journal.pntd.0001407 (PMC3226457; doi:10.1371/journal.pntd.0001407)
Supplement: Table S1 — Current and projected climatic suitability for Phlebotomus species in Central Europe. Noted are mean values and standard deviation in brackets. Projections refer to the B1 scenario. (DOC) [file pntd.0001407.s003.doc]

Table S1: Current and projected climatic suitability for *Phlebotomus* species in Central Europe. Noted are mean values and standard deviation in brackets. Projections refer to the B1 scenario.

|  | Central Europe | | | | Austria | | | | Germany | | | | Switzerland | | | |
| --- | --- | --- | --- | --- | --- | --- | --- | --- | --- | --- | --- | --- | --- | --- | --- | --- |
|  | current | 2011-2040 | 2041-2070 | 2071-2100 | current | 2011-2040 | 2041-2070 | 2071-2100 | current | 2011-2040 | 2041-2070 | 2071-2100 | current | 2011-2040 | 2041-2070 | 2071-2100 |
| *P. ariasi* | 0.14 (+/- 0.10) | 0.26 (+/- 0.10) | 0.32 (+/- 0.15) | 0.42 (+/- 0.16) | 0.02 (+/- 0.02) | 0.19 (+/- 0.18) | 0.21 (+/- 0.18) | 0.30 (+/- 0.21) | 0.17 (+/- 0.08) | 0.28 (+/- 0.10) | 0.36 (+/- 0.11) | 0.48 (+/- 0.09) | 0.07 (+/- 0.08) | 0.19 (+/- 0.22) | 0.20 (+/- 0.23) | 0.21 (+/- 0.23) |
| *P. mascittii* | 0.28 (+/- 0.19) | 0.34 (+/- 0.20) | 0.36 (+/- 0.22) | 0.38 (+/- 0.22) | 0.26 (+/- 0.17) | 0.21 (+/- 0.20) | 0.21 (+/- 0.19) | 0.22 (+/- 0.20) | 0.32 (+/- 0.18) | 0.33 (+/- 0.12) | 0.37 (+/- 0.15) | 0.46 (+/- 0.17) | 0.21 (+/- 0.18) | 0.10 (+/- 0.10) | 0.12 (+/- 0.16) | 0.13 (+/- 0.17) |
| *P. perniciosus* | 0.25 (+/- 0.14) | 0.30 (+/- 0.16) | 0.36 (+/- 0.18) | 0.42 (+/- 0.20) | 0.09 (+/- 0.10) | 0.16 (+/- 0.17) | 0.19 (+/- 0.19) | 0.23 (+/- 0.21) | 0.31 (+/- 0.10) | 0.36 (+/- 0.12) | 0.43 (+/- 0.12) | 0.49 (+/- 0.12) | 0.13 (+/- 0.15) | 0.09 (+/- 0.14) | 0.11 (+/- 0.17) | 0.14 (+/- 0.21) |
|  |  |  |  |  |  |  |  |  |  |  |  |  |  |  |  |  |
| *P. neglectus* | 0.23 (+/- 0.10) | 0.28 (+/- 0.12) | 0.36 (+/- 0.15) | 0.38 (+/- 0.14) | 0.18 (+/- 0.13) | 0.25 (+/- 0.17) | 0.33 (+/- 0.20) | 0.36 (+/- 0.20) | 0.24 (+/- 0.08) | 0.29 (+/- 0.07) | 0.37 (+/- 0.11) | 0.38 (+/- 0.10) | 0.23 (+/- 0.17) | 0.28 (+/- 0.25) | 0.40 (+/- 0.30) | 0.41 (+/- 0.29) |
| *P. perfiliewi* | 0.10 (+/- 0.06) | 0.16 (+/- 0.15) | 0.15 (+/- 0.15) | 0.21 (+/- 0.18) | 0.04 (+/- 0.03) | 0.20 (+/- 0.23) | 0.22 (+/- 0.25) | 0.27 (+/- 0.28) | 0.12 (+/- 0.05) | 0.15 (+/- 0.11) | 0.14 (+/- 0.10) | 0.19 (+/- 0.14) | 0.05 (+/- 0.04) | 0.13 (+/- 0.21) | 0.11 (+/- 0.20) | 0.17 (+/- 0.24) |
